# Syphilitic gumma presenting as squamous cell carcinoma of Vulva: A case report

**DOI:** 10.1016/j.gore.2024.101478

**Published:** 2024-08-08

**Authors:** R.M. Nuss, A.J. Lazenby, H.C. Provost, T. Castellano

**Affiliations:** a University of Oklahoma at Tulsa, Department of Obstetrics and Gynecology, USA; b Louisiana State University, New Orleans, Department of Obstetrics and Gynecology, USA; c Louisiana State University, Lafayette Campus, Department of Obstetrics and Gynecology, USA; d Louisiana State University, New Orleans, Division of Gynecologic Oncology, USA

**Keywords:** Case Report, Gumma, Syphilis, Vulva

## Abstract

**Introduction:**

Tertiary syphilis, a late form of the disease with neurologic, cutaneous, and cardiovascular manifestations, is rarely seen in the United States in modern times. Also called the “great imitator,” syphilis tends to mimic other disease processes, which can lead to a delay in proper diagnosis and treatment.

**Case:**

We present the case of a 59-year-old woman who presented for evaluation of possible squamous cell carcinoma of the vulva. After multiple inconclusive biopsies and extensive workup with multiple providers, she was instead found to have cutaneous and neurologic tertiary syphilis. A correct diagnosis was delayed in this patient due to a presentation not consistent with classical teachings and due to a lack of access to care.

**Conclusion:**

Syphilis has characteristic signs and symptoms, but in practice, it can have an indolent presentation that may differ from traditional presentations. To our knowledge, this is the first comprehensive case report of vulvar cutaneous syphilis masquerading as squamous cell carcinoma of the vulva. This case also provides additional evidence for the necessity of comprehensive gynecologic care and sexual history-taking in the post-menopausal populations and in rural communities.

## Highlights

• Syphilis, also known as the “Great Imitator,” has historically been misdiagnosed as other diseases.
• In modern times, tertiary syphilis is rarely seen, which can lead to diagnostic confusion.
• Tertiary syphilis may present in ways not consistent with traditional descriptions of the disease.
• Tertiary cutaneous syphilis can masquerade as a painful, rapidly-growing squamous cell carcinoma of the vulva.
• Rising rates of primary symphilis emphasize the importance of characterizing atypical presentations of tertiary syphilis.

## 1 Introduction

Tertiary syphilis generally develops more than three years after initial infection with the spirochete *Treponema pallidum* subspecies *pallidum*. It occurs in about one third of patients with untreated syphilis worldwide (Moon et al., 2018). Despite the high prevalence in the world, this late presentation is rarely seen in the developed world. Tertiary syphilis typically presents as cutaneous (gummatous) syphilis, neurosyphilis, and cardiovascular syphilis (French, 2007 Jan 20). The manifestations result from spirochetes invading the endothelial cells of blood vessels in different parts of the body (Xie et al., 2022). Gummas, classically taught to be “non-tender nodular lesions with central punched-out necrosis” (Moon et al., 2018) that result from years of inflammation due to the presence of spirochetes, are the hallmark of cutaneous syphilis. The lesions are not consistent in appearance and tend to mimic other disease processes, which can lead to initial misdiagnosis and delay in treatment (Yan et al., 2022 May). Fortunately, manifestations of advanced disease can be prevented with adequate early treatment. If more advanced stage syphilis is found, it should be treated immediately to prevent the outcomes above (Meyers et al., 2008 Mar 15). Following treatment, most patients make a full recovery from their symptoms (Gong et al., 2022 May).

We describe an atypical case of cutaneous syphilis that mimicked squamous cell carcinoma of the vulva (VSCC).

## 2 Case

Our patient is a 59-year-old post-menopausal woman who presented with a right-sided rapidly growing, painful vulvar mass. The patient had minimal medical history due to limited access to the medical system outside of obstetric care. Menopause occurred 10 years prior to presentation, and she denied any post-menopausal bleeding. Social history was notable for a 17-pack year history. Last sexual activity was about a year prior to her presentation, and she reported having numerous male and female partners without consistent protection over the last decade.

The chief complaint was a painful lump in her groin, which she attributed to a bug bite or gardening injury. However, the mass persisted for several days, and her pain intensified, prompting presentation to her local emergency department where she was diagnosed with cellulitis and given analgesics, topical ointment, and a 7-day course of trimethoprim/sulfamethoxazole. The patient presented again three days later without improvement, and she was instructed to finish her course of antibiotics and referred to gynecology. Two weeks later, the gynecologist collected a pap smear and three vulvar punch biopsies and referred the patient to gynecologic oncology with concern for VSCC. Cervical cytology and high-risk HPV testing were negative. Pathology demonstrated ulcerated squamous mucosa with adjacent epidermal hyperplasia, dermal inflammation, and focal epithelial atypia. These findings could be seen with malignancy but were not diagnostic of VSCC.

The following month at the gynecologic oncology clinic, the patient stated that her vulvar symptoms had gotten progressively worse and were now associated with significant pain, swelling, drainage, and inability to sit. She also reported a daily headache and fatigue. She denied other constitutional symptoms including fever, weight loss, and chills. The pelvic exam at this time showed a 3.5 x 2 cm right labia minora ulcerated friable lesion, a 1.5 x 2.5 cm friable area superior to the primary lesion, and a 1 x 1 cm left vulvar lesion with ulceration. Lesions showed no urethral, vaginal, clitoral, or anal involvement. Groin exam showed 1–2 cm inguinofemoral lymphadenopathy. The patient was unable to tolerate another in-clinic biopsy, so further laboratory testing was ordered. Due to concern for vulvar cancer with metastasis, a plan was made for a bilateral modified vulvectomy with inguinofemoral lymph node dissection. As is routine, a full STI panel with HIV, hepatitis and RPR was obtained for surgical planning.

One week later, the patient contacted the clinic to report worsening headache and fatigue now associated with upper respiratory symptoms. Surgery was deferred, and the patient underwent additional vulvar biopsies. Initial pathologic evaluation of the biopsies showed skin with pseudoepitheliomatous hyperplasia overlying granulomatous and suppurative inflammation with neutrophil abscesses accompanied by many plasma cells. No microorganisms were highlighted by Gram, PAS, Warthin starry or T. Pallidum stains. However, her lab results soon returned with an RPR titer of 1:64 and subsequent positive *Treponema pallidum* antibody was found. The biopsy samples were reassessed due to this new clinical information and findings included plasma cell infiltrates and psoriaform hyperplasia, which are common to spirochete infections. A spirochete immunohistochemical stain was done and focally positive for cutaneous syphilis. Additional biopsies confirmed the diagnosis of tertiary syphilis and ruled out comorbid VSCC. A series of three bicillin injections was initiated to address the patient’s cutaneous syphilis.

After her first injection, she was seen by infectious disease (ID) and continued to report brain fog, fatigue, and headaches. Due to concern for neurosyphilis, ID ordered a computed tomography (CT) scan of the head and performed a lumbar puncture. Her head CT demonstrated nonspecific white matter microvascular changes. Lumbar puncture was notable for negative VDRL, elevated white blood cell count, and elevated protein. Diagnostic criteria include CSF white blood cell count greater than 20, a reactive CSF VDRL, or a positive intrathecal T pallidum antibody index. In conjunction with her headaches, visual changes and fatigue, this result ultimately garnered the diagnosis and subsequent treatment for neurosyphilis.

**Fig. 1:**
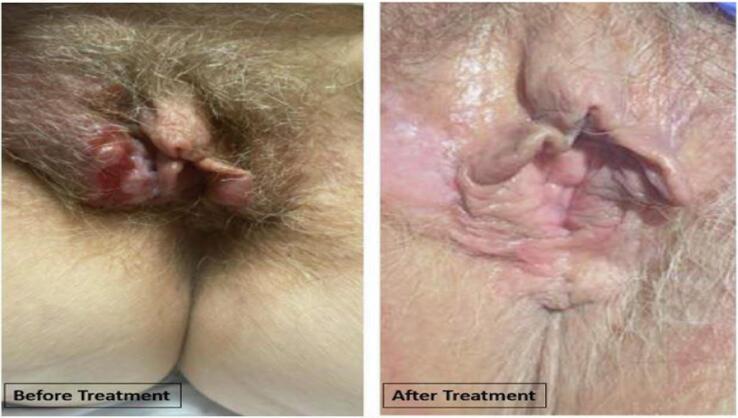
Clinical pictures pre- and post-treatment.

**Fig. 2:**
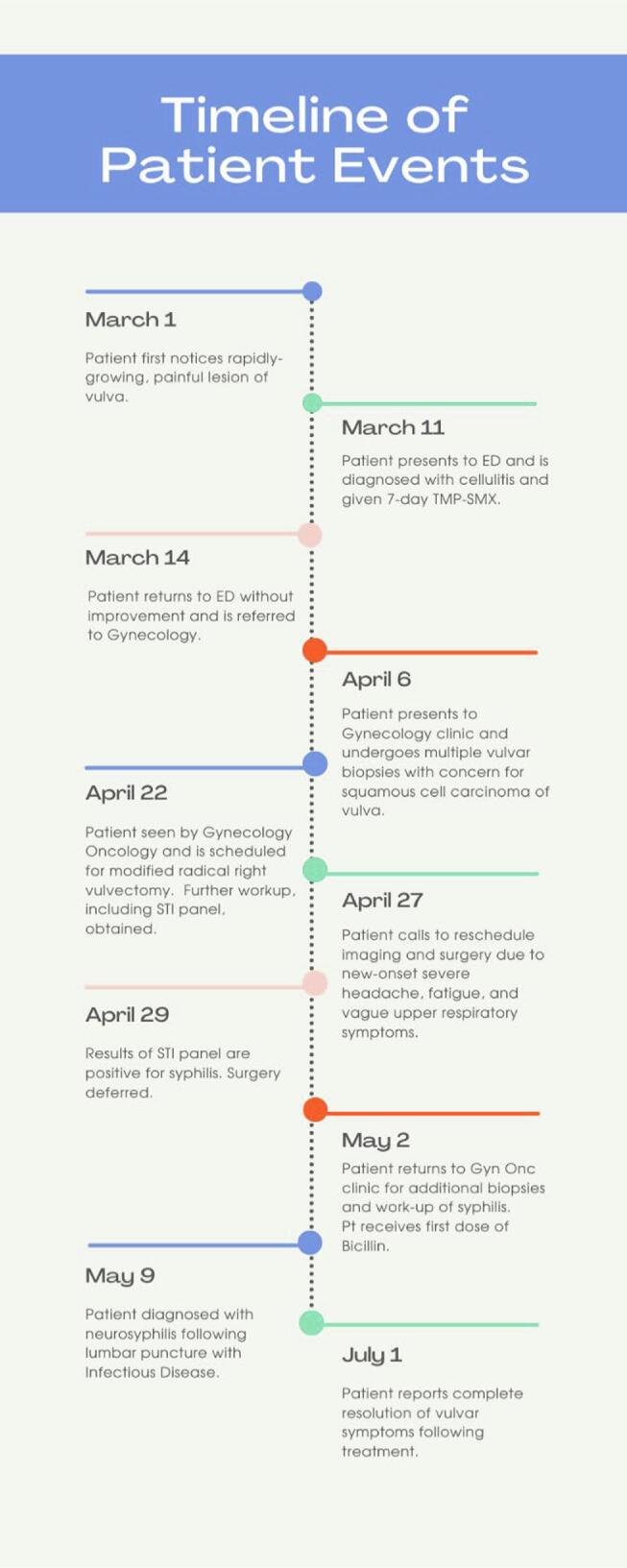
Timeline of patient events.

Over the next month following treatment, the patient’s vulvar masses decreased significantly in size and severity (Fig. 1), and her neurologic symptoms resolved. The patient reported she was able to return to her normal activities of daily life including gardening and biking.Fig. 2..

## 3 Discussion

Although syphilis is known as the ‘great imitator’, it more commonly masquerades as other infectious disease processes rather than malignancy (Solis et al., 2018 Dec). The concern for VSCC to rapid, tumor-like growth is what makes this case unique.

Cutaneous syphilis is exceedingly rare in the United States. However, new cases of VSCC are diagnosed in approximately 6,000 women in the United States every year and are associated with significant morbidity and mortality (Singh and Gilks, 2020 Jan). VSCC most commonly presents with vulvar pruritis, palpable mass, and vulvar bleeding (Gynecologic cancers). The presence of bilateral inguinofemoral lymphadenopathy in the context of a rapidly growing mass in a 59-year-old patient raised immediate suspicion for VSCC. In addition, multiple lifetime sexual partners and inconsistent gynecologic care left questions about the presence of HPV. Characteristics of our patient’s vulvar mass including ulceration, drainage, and pain are all seen in typical presentations of VSCC (Gynecologic cancers).

Standard of care in patients with suspected VSCC includes obtaining biopsies of the lesion to confirm pathologic diagnosis and completing surgical staging to determine course of treatment (Gynecologic cancers). Due to the rapidly progressive nature of her lesion, inconclusive biopsies, and significant symptom burden, she was scheduled for expedited modified radical vulvectomy pending further work-up and possible intraoperative frozen section. In addition, the patient’s presentation with a painful lesion was not consistent with the classical teaching that cutaneous gummas are painless growths. Fortunately, the patient’s screening panel for sexually transmitted infections revealed her syphilitic infection prior to her surgery. The STI workup was the factor that ultimately contributed to making the correct diagnosis. Though this was obtained due to the concern for an HPV associated malignancy, it ultimately led the gynecologic team to expand the differential diagnosis and coordinate with other subspecialties to obtain appropriate tissue testing to confirm diagnosis and rule out malignancy.

In accordance with 2016 United States Preventative Services Task Force (USPSTF) guidelines, high-risk nonpregnant women aged 25 or greater should be screened for syphilis. Women in the high-risk population are those with history of HIV infection, history of incarceration, history of commercial sex work, certain racial/ethnic groups, and residence in an area with high rates of syphilis (Recommendation | United States Preventive Services Taskforce). These guidelines are currently being updated. Our patient’s residence in Lafayette Parish, Louisiana, which has a significant syphilis rate of 7 cases per 100,000 as of 2020 (LDH Department of Public Health. Region 4- lafayette STD/HIV update., 2019), did qualify her for screening. However, she unfortunately had few episodes of interface with the healthcare system and was lacking in several preventative healthcare maintenance items. In cases that deviate from guidelines, a physician's clinical judgement remains of the utmost importance. Regular care with a gynecologist and a quality sexual history likely would have prevented progression to tertiary syphilis in this patient.

Late-stage syphilis has been almost entirely eradicated in developed countries (Moon et al., 2018). The presence of tertiary syphilis, therefore, tends to be in patients without access to routine preventative healthcare (French, 2007 Jan 20). This patient rarely interfaced with the healthcare system throughout her life, resulting in her presentation in advanced stage disease. This presents two public health concerns: prevention of adverse health outcomes in the individual and prevention of infectious spread in the community. Syphilis rates in the United States have increased by 80 % from 2018 to 2022 which may foreshadow an increase in the number of tertiary syphilis cases diagnosed in the population (2022). Tertiary syphilis is not contagious; however, the presence of tertiary syphilis implies that primary and secondary syphilis, which are contagious, went untreated in an individual. As a result, this patient had the potential to unknowingly spread syphilis to others in her community.

In conclusion, this case illustrates a rare case of vulvar syphilis mistaken for gynecologic malignancy. The diagnostic process was complicated in this patient by a rare manifestation of syphilis not consistent with classical teachings and by a lack of access to care. This highlights the importance of initiatives to increase access to care in underserved rural areas. As the prevalence of sexually transmitted infections in the Deep South continues to rise, physicians should make a point to take comprehensive sexual histories in all patients, regardless of factors like age, race/ethnicity, and socioeconomic status. In addition, when evaluating a rapidly progressive vulvar mass, a syphilitic gumma is a rare but important component in the differential diagnosis.

## 4 Author contribution Statement

A.L., H.P., and T.C. participated in the care of this patient and presented the idea to write up this case report. T.C. encouraged R.N. to perform a literature review around tertiary syphilis and vulvar squamous cell carcinoma. R.N. wrote an initial draft which was sent to A.L., H.P., and T.C who edited and contributed their expertise. R.N. made necessary revisions based on their feedback. Under the supervision of T.C., all authors helped shape the final draft of this manuscript.

## 5 Informed Consent Statement

Written informed consent was obtained from the patient for publication of this case report and accompanying images.

## CRediT authorship contribution statement

**R.M. Nuss:** Writing – review & editing, Writing – original draft. **A.J. Lazenby:** Writing – review & editing, Conceptualization. **H.C. Provost:** Writing – review & editing, Conceptualization. **T. Castellano:** Writing – review & editing, Supervision, Conceptualization.

## References

[b0005] 2022 U.S. syphilis cases reach highest numbers since the 1950s. Centers for Disease Control and Prevention. January 30, 2024. https://www.cdc.gov/nchhstp/newsroom/releases/2024/STI-Surveillance-Report-2022.html#:∼:text=Reported%20syphilis%20cases%20increased%2080,blindness%2C%20deafness%2C%20and%20paralysis.

[b0015] French P. (2007 Jan 20). Syphilis. BMJ..

[b0020] Gong H.Z., Li J., Zheng H.Y. (2022 May). The treatment outcome and predictors of serological response in syphilis in a sexually transmitted infections center. China. Int J STD AIDS..

[b0025] (2013). Reference & Research Book News..

[b0030] LDH Department of Public Health. Region 4- lafayette STD/HIV update. 2019.

[b0035] Meyers D., Wolff T., Gregory K., Marion L., Moyer V., Nelson H., Petitti D., Sawaya G.F. (2008 Mar 15). Uspstf. USPSTF recommendations for STI screening. Am Fam Physician..

[b0040] Moon J., Yu D.A., Yoon H.S., Cho S., Park H. (2018). Syphilitic Gumma: A Rare Form of Cutaneous Tertiary Syphilis. Annals of Dermatology..

[b0045] Recommendation | United States Preventive Services Taskforce [Internet]. www.uspreventiveservicestaskforce.org. Available from: https://www.uspreventiveservicestaskforce.org/uspstf/recommendation/syphilis-infection-in-nonpregnant-adults-and-adolescents.

[b0050] Singh N., Gilks C.B. (2020 Jan). Vulval squamous cell carcinoma and its precursors. Histopathology..

[b0055] R.N. Solis B.T. Kuhn D.G. Farwell An Unusual Case of Tertiary Syphilis Behaving Like Tongue Squamous Cell Carcinoma J Investig Med High Impact Case Rep. 20 6 2018 Dec 2324709618820355 10.1177/2324709618820355. PMID: 30622992; PMCID: PMC6302270. 10.1177/2324709618820355 PMC6302270 30622992

[b0060] Xie B, Zhao T, Zhao S, Zhou J, Zhao F. Possible effects of Treponema pallidum infection on human vascular endothelial cells. J Clin Lab Anal. 2022 Apr;36(4):e24318. doi: 10.1002/jcla.24318. Epub 2022 Mar 10. PMID: 35274369; PMCID: PMC8993650. 10.1002/jcla.24318 PMC8993650 35274369

[b0065] Yan J., Luo L., Han J., Yan D., Zhang B., Zhang Z., Shi J., Zhu M., Yu J., Liu S., Qi J., Yang Z. (2022 May). Comparing Noninvasive Predictors of Neurosyphilis Among Syphilis Patients With and Without HIV Co-Infection Based on the Real-World Diagnostic Criteria: A Single-Center, Retrospective Cohort Study in China. AIDS Res Hum Retroviruses..

